# Osteopetrosis misdiagnosed as congenital cytomegalovirus infection: A case report and literature review

**DOI:** 10.1097/MD.0000000000045583

**Published:** 2025-11-07

**Authors:** Xue Jin, Wei Wang, Zhen Pan, Mingyan Jiang, Yu Zhu

**Affiliations:** a Department of Pediatrics, West China Second University Hospital, Sichuan University, Chengdu, China; b West China School of Medicine, Sichuan University, Chengdu, China; c The Second People’s Hospital of Yibin, Yibin, China; d Key Laboratory of Birth Defects and Related Diseases of Women and Children (Ministry of Education), Sichuan University, Chengdu, China.

**Keywords:** congenital cytomegalovirus infection, hearing and optic nerve abnormalities, hepatosplenomegaly, infantile malignant osteosclerosis, thrombocytopenia

## Abstract

**Rationale:**

Infantile malignant osteopetrosis (IMO) (OMIM 259700) is a rare autosomal recessive disease that is caused by defective function of osteoclasts or a reduced number of osteoclasts, resulting in extensive bone sclerosis. The morbidity and mortality rates are extremely high. It may have similar clinical manifestations with congenital cytomegalovirus infection. Due to limited knowledge and heterogenous manifestations, clinical early diagnosis of the disease is challenging.

**Patient concerns:**

We reported a case of a 3-month, 15-day-old female child who presented with hematologic abnormalities, hepatosplenomegaly, and optic nerve abnormalities. The child was misdiagnosed congenital cytomegalovirus at the initial diagnosis. However, the child’s condition did not improve significantly after antiviral treatment. Fortunately, the child was then genetically tested for bone marrow failure disorders.

**Diagnoses:**

Infantile malignant osteopetrosis.

**Interventions:**

The treatment involved ganciclovir and transfusion of blood products. However, after 3 weeks of antiviral treatment, the child did not improve significantly. At that time, bone marrow failure disease and immunodeficiency disease gene test report suggested IMO. Her parents refused to do hematopoietic stem cell transplantation.

**Outcomes:**

Oral valganciclovir and transfusion of blood products treatment was continued.

**Lessons:**

Our report expands the understanding of IMO disease. It is important that patients with clinical features and hematologic findings similar to infection should undergo a thorough clinical evaluation and timely genetic analysis to identify the causative mutation to avoid delays in diagnosis and treatment.

## 1. Introduction

Congenital cytomegalovirus (cCMV) infection is a common congenital infection that occurs in 1/100–200 live births worldwide. Approximately 10% of children who develop nonspecific signs and symptoms, such as hepatosplenomegaly, seizures or intracranial abnormalities, and sensorineural hearing loss (unilateral or bilateral).^[1]^ Some children have blood abnormalities, including increased leukocytes, anemia, decreased platelets, and blast cells in the peripheral blood in symptomatic congenital cytomegalovirus infection at birth.^[2]^ Infantile malignant osteopetrosis (IMO) can be clinically similar to cCMV infection in its early stages, making it easy to misdiagnose. IMO is the most severe type of osteosclerosis and has the fastest disease progression. The incidence rate is 1:200,000.^[3]^ Approximately 50% of cases are due to double allele mutations in T-cell immune regulator 1 (*TCIRG1*).^[4]^ The gene encoding the A3 subunit of osteoclast V-ATPase is the basis for the establishment of an acidic environment in the resorption region of osteoclasts.^[5]^ The typical clinical manifestations of IMO are generalized osteosclerosis, pathologic fractures, cranial nerve entrapment (predominantly the optic nerve), bone marrow failure, hepatosplenomegaly, and recurrent infections. The diagnosis of IMO is mainly based on genetic testing and specific imaging changes. Allogeneic hematopoietic stem cell transplantation (HSCT) is the only curative treatment for IMO.^[6]^ However the correlation of cCMV infection and Osteopetrosis is unclear. Pediatricians are prone to misdiagnose IMO as cCMV infection due to a lack of knowledge about IMO. Therefore the purpose of this case report is to identify the heterogeneity of the clinical presentation of IMO to avoid delayed diagnosis and treatment and to enrich the database of *TCIRG1* gene mutations.

## 2. Case report

**Figure 1.**
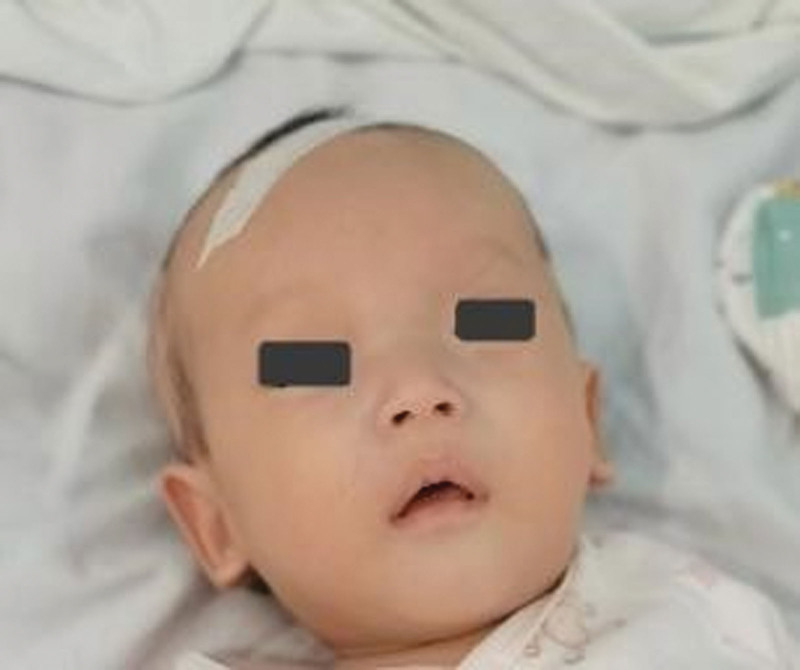
Note frontal bossing.

The patient was a 3-month and 15-day-old female infant admitted to the pediatric department with abnormal blood counts and hepatosplenomegaly for more than 1 month, G1P1. Her parents denied a consanguineous marriage. At the time of admission, her body weight was 4.8 kg (<P3), length was 55cm (<P3), with a head circumference of 39 cm (<P25), poor visual and vocal tracking, prominent forehead, bulging fontanel, nystagmus, sensitive pupil light reflex, no lymph node enlargement, mild pale, and no rashes, ecchymosis, or petechiae. The liver was 7 cm below the ribs and 2 cm below the xiphoid process. The spleen was 7 cm in line I, 7 cm in line II, and 2 cm in line III. Neck resistance was negative. Muscle tone in the limbs was reduced. Primitive reflexes were smoothly elicited (Fig. 1).

The white blood cell count was 23.8 × 10^9^/L (normal range 4.3–14.2 × 10^9^/L), the blast cell count was 1.14 × 10^9^/L (normal range 0), the monocyte count was 1.7 × 10^9^/L (normal range 0.15–1.56 × 10^9^/L), the hemoglobin level was 67 g/L (normal range 97–183 g/L), and the platelet count was 16 × 10^9^/L (normal range 151–536 × 10^9^/L). The percentage of reticulocytes was 13.17%, and the percentage of reticulocytes was 0.4149 × 10^12^/L (normal range 0.024–0.084 × 10^12^/L). The alkaline phosphatase level was 1631 U/L (normal range 125–250 U/L). Calcium and phosphorus metabolism, vitamin D, thyroid function, cellular immunity, humoral immunity, antihuman globulin test results, hemoglobin electrophoresis results and glucose 6-P dehydrogenase results were normal. The purified protein derivative results of the tuberculin skin test, T-cell spot test, and Epstein–Barr virus nucleic acid test were negative. The serum CMV nucleic acid concentration was 1.25 × 10^3^/mL with reverse transcription polymerase chain reaction. Both the patient’s urine and her mother’s breast milk were positive for CMV nucleic acid. But CMV nucleic acid of dried blood spot was not performed in 3 weeks after birth, and the mother was not tested for CMV nucleic acid during pregnancy. Visual evoked potentials indicated that visual organ–visual cortex conduction dysfunction could not be excluded. Screening for retinopathy revealed no abnormalities in the optic disc or macular structures or retinal abnormalities. Electroencephalogram indicated boundary infancy electroencephalogram, including several occipital spikes and fast waves during sleep. Nevertheless, clinical episodes identified by her parents were monitored without electroencephalogram changes. Bone marrow examination revealed that the granulocyte–red blood cell ratio was small, with 3.0% blast lymphocytes. The juvenile myelomonocytic leukemia (JMML) diagnostic and prognostic gene panels revealed no class I variants. The *BCR::ABL* fusion gene was negative.

**Figure 2.**
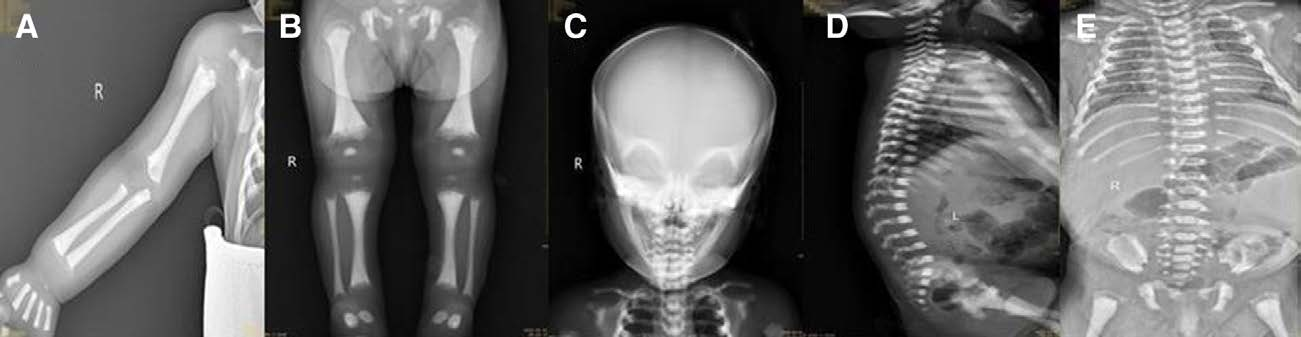
Radiology images of the patient demonstrating diffuse increased bone density. (A) Humerus, ulna, and radius. (B) Bilateral tibiae, iliac wings, and long bones. (C) Skull. (D) Vertebrae. (E) Bilateral ribs.

At the beginning of the diagnosis and treatment, the child underwent thoracic and abdominal computed tomography and cranial magnetic resonance imaging. Unfortunately, the IMO imaging changes were not recognized early. After the child’s genetic testing revealed IMO, a second plain radiograph of the extremities, skull, and vertebrae was taken, and it suggested a diffuse increase in bone density (Fig. 2).

**Figure 3.**
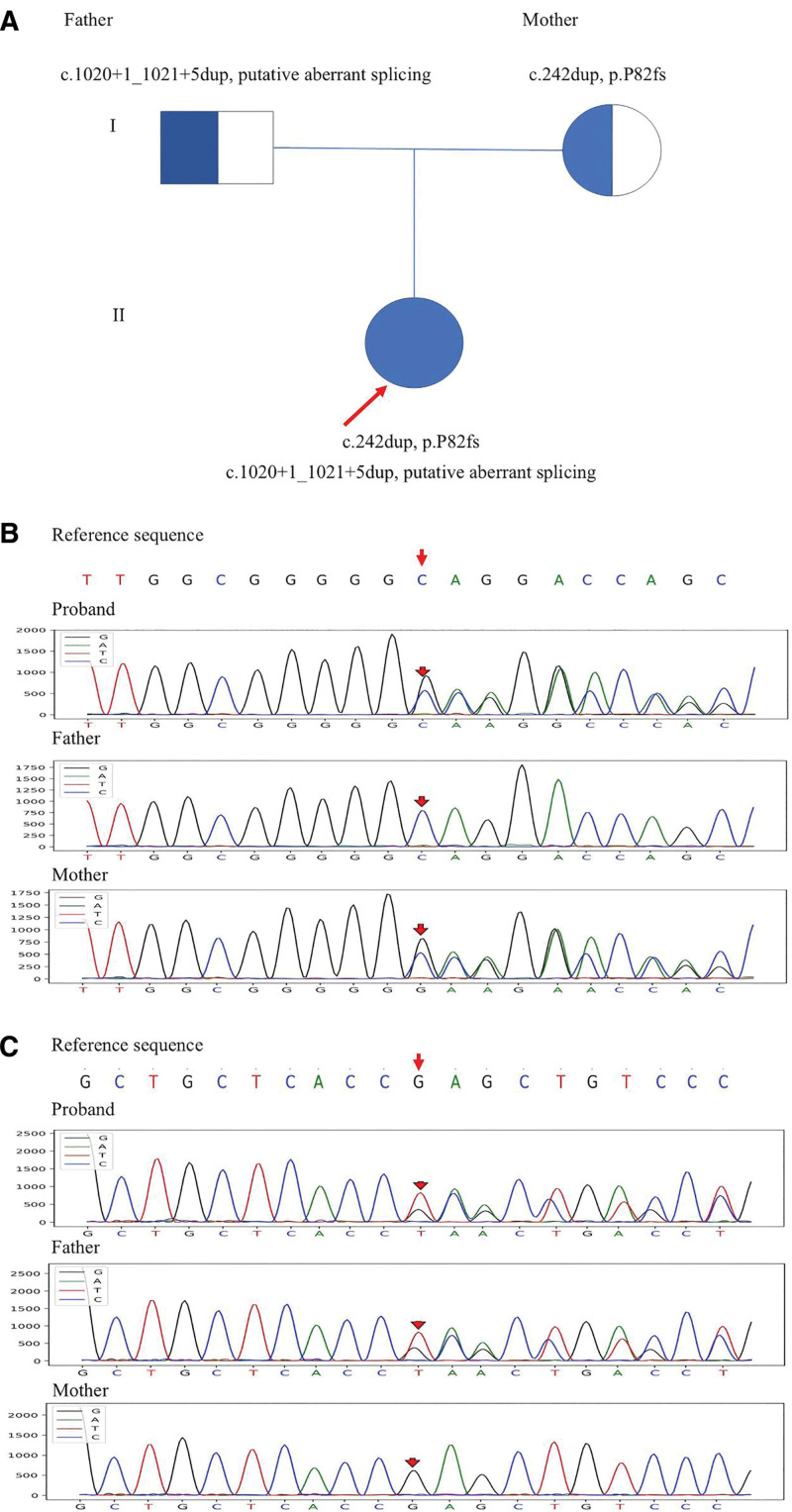
Family tree and sanger sequencing maps. (A) None of the parents had similar symptom manifestations. (B) T-cell immune regulator 1, c.242dup, heterozygous mutation at chr11:67810150 were found in both the patient and her mother. (C) T-cell immune regulator 1, c.1020 + 1_1021 + 5dup heterozygous mutation at chr11:67811810 were found in both the patient and her father. The proband is marked with long red arrow. The short red arrows indicate the start positions of the mutations.

The patient was suspected of having JMML due to increased peripheral blood leukocytes and monocytes with anemia, decreased platelets, and hepatosplenomegaly, but this was ruled out by bone marrow examination and JMML-related genetic testing. Moreover, the child was found to be positive for blood and urine CMV nucleic acid, in combination with early onset of disease, a small head circumference, optic nerve involvement, hepatosplenomegaly and hematologic involvement, and was treated with ganciclovir and transfusion of blood products. However, after 3 weeks of antiviral treatment, the child did not improve significantly. And there was no hearing loss. At that time, bone marrow failure disease and immunodeficiency disease gene test results suggested IMO. The results revealed that the infant had heterozygous mutations in the *TCIRG1* gene. A frameshift variant c.242dup in exon 4 was originated from her mother (NM_006019) and the c.1020 + 1_1021 + 5dup variant in exon 9 was inherited from her father (NM_006019). The double heterozygous variants resulted in a frameshift at amino acid 82 (p.P82fs) and a putative aberrant splicing respectively, generating a premature truncation. However, none of the parents had similar symptom manifestations (Fig. 3). A cranial/limb/spinal plain X-ray film suggested diffuse bone thickening of the whole body. Combined with the clinical manifestations, imaging examinations and genetic tests, the child was finally diagnosed with IMO (autosomal recessive osteosclerosis type 1). The parents were informed of the patient’s diagnosis and prognosis. Oral valganciclovir treatment was continued, but her parents refused HSCT.

## 3. Discussion

Osteopetrosis is a heterogeneous group of disorders, which are reflected in the causative genes, mode of inheritance, clinical presentation and disease severity. Approximately 50% of cases of IMO are due to *TCIRG1* double allele mutations, and 17.5% are due to *CLCN7* double allele mutations. Other rare causative genes are *SNX10, OSTM1, TNFRSF11A*, and *TNFSF11.*^[7]^ In this study, 2 mutations in *TCIRG1* were reported for the first time, namely, c.242dup:p. (Pro82Alafs*56) and c.1020–1021:p. (Met341Valfs*7), but the functions and regulatory mechanisms of these 2 mutations need to be further investigated. The phenotype of IMO has a wide range of clinical manifestations and lacks gene–phenotype correlations. However, a related study revealed a statistically significant correlation between *TCIRG1* mutation and thrombocytopenia, suggesting that this gene mutation was more aggressive.^[8]^

The clinical presentation is diverse, and the diagnosis is challenging. Cytomegalovirus is a weak pathogen with latent-activating biological properties. It is a common pathogen causing diseases in fetuses and neonates with developmental immunodeficiencies. It also shares clinical features with IMO (Table 1), including pancytopenia, hepatosplenomegaly, fetal growth retardation, sensorineural hearing impairment and intracranial calcification. Obviously, differentiating between these 2 diseases on the basis of their clinical manifestations is very difficult. In this case, CMV infection was detected in the early stages of diagnosis and treatment. However, it was very regrettable that CMV nucleic acid test of of dried blood spot was not performed within 3 weeks after birth, and the mother was not tested for CMV nucleic acid during pregnancy. But intraocular and intracranial CMV infections are mostly congenital. cCMV infection was not excluded in the patient with multiple organ damage and no immune deficiency. It was debatable whether blood and neurological involvement weree related to cytomegalovirus infection because there was no early postnatal etiological evidence and hearing loss. Imaging examination is beneficial for distinguishing between these 2 diseases. It is not known whether IMO increases susceptibility to CMV infection. To date, only 2 cases of combined CMV infection with IMO had been reported,^[9,10]^ but the relationship between congenital CMV infection and osteosclerosis was not described. CMV infection was only found during screening for the cause of abnormal blood pattern with hepatosplenomegaly. We suspect that osteosclerosis may lead to bone marrow hematopoietic failure and production of immune cells, which increases the risk of infection. So it should be reported more often in the future to better determine the significance of CMV infection in IMO.

**Table 1:** cCMV infection compared with IMO: clinical, imaging, and laboratory findings.

IMO	cCMV
Symptoms and signs	Symptoms and signs
Short stature	At birth:
Skin changes	Petechiae/purpura
Optic atrophy	Jaundice
Macrocephaly	Hepatosplenomegaly
Developmental delay	Seizures
Convulsions	Small for gestational age
Recurrent infections	Microcephaly
Anemia/pancytopenia	Central nervous system involvement
Hepatosplenomegaly	Sensorineural hearing loss at birth
Dental problems	Chorioretinitis
Fractures	Long term sequelae:
Delayed fracture healing	Any neurodevelopmental impairment
Laboratory test	Intellectual disability
Leukocytosis	Borderline intelligence
Anemia	Delayed onset sensorineural hearing loss
Thrombocytopenia	Any Sensorineural hearing loss
Hypercalcemia	Ophthalmic abnormality
Imaging	Vision impairment
X-ray	Legal blindness
Diffuse increased bone density (marble bone appearance)	Laboratory tests
Erlenmeyer flask deformity	Thrombocytopenia
Bone-within-a-bone	Elevated transaminases or bilirubin
Sandwich vertebrae	CMV viral load
	Imaging
	MRI: evidence of white or gray matter changes, including cysts, and migration defects such as polymicrogyria
	Cranial ultrasound: calcification or ventriculomegaly
	Ophthalmology
	Chorioretinitis, optic atrophy, cataracts
	Hearing test
	Auditory brainstem response: sensorineural hearing loss

cCMV = congenital cytomegalovirus, IMO = infantile malignant osteopetrosis.

Malignant infantile osteopetrosis is a rare and severe autosomal recessive ossification disorder. It can present early in life and is often fatal without appropriate treatment. Early diagnosis is the key to proper treatment. The diagnosis of IMO is dependent on imaging, and for timely diagnosis of IMO, we recommend early genetic testing, such as whole-exome sequencing. The only curative treatment to date for IMO is HSCT, and IMO is usually fatal within the first 10 years of life if not be treated. Despite the outcome of IMO is greatly improved via HSCT, alternative autologous therapies such as hematopoietic stem cell-targeted gene therapy have the potential to restore the resorptive function of osteoclasts without some of the complications associated with allogeneic HSCT such as limited availability of a matching donor and graft-versus-host disease.^[11]^ Based on this growing body of safety data, gene therapy utilizing autologous hematopoietic stem and progenitor cells therefore represents a potentially safe and promising therapeutic alternative for IMO gene therapy.^[12]^

## 4. Conclusion

In summary, for the first time, we reported 2 *TCIRG1* gene mutations, which enrich the database of *TCIRG1* gene mutations. We suggest careful differential diagnosis, comprehensive clinical evaluation and imaging of patients with clinical features and laboratory findings similar to those of cytomegalovirus infection to improve the understanding of IMO. Early performance of relevant genetic tests can help provide more timely and accurate guidance.

## Acknowledgments

The authors are grateful to the patient and her parents for their courageous battle against the disease and for their invaluable contribution to this study.

## Author contributions

**Conceptualization:** Mingyan Jiang, Xue Jin, Wei Wang, Zhen Pan, Yu Zhu.

**Investigation:** Mingyan Jiang, Xue Jin, Wei Wang, Yu Zhu.

**Formal analysis:** Xue Jin, Wei Wang, Zhen Pan.

**Funding acquisition:** Yu Zhu.

**Methodology:** Mingyan Jiang, Yu Zhu.

**Project administration:** Mingyan Jiang, Xue Jin, Wei Wang, Yu Zhu.

**Resources:** Mingyan Jiang, Yu Zhu.

**Supervision:** Mingyan Jiang, Yu Zhu.

**Validation:** Mingyan Jiang, Yu Zhu.

**Visualization:** Mingyan Jiang, Yu Zhu.

**Writing – review & editing:** Mingyan Jiang, Yu Zhu.

**Writing – original draft:** Xue Jin, Wei Wang.
